# Real-world data on early initiation of sodium-glucose co-transporter-2 inhibitors in newly diagnosed heart failure with reduced ejection fraction

**DOI:** 10.1093/eschf/xvag008

**Published:** 2026-02-17

**Authors:** Masatake Kobayashi, Luca Monzo, Guillaume Baudry, Gema Hernandez, Olivier Denquin, Kevin Duarte, Nicolas Girerd

**Affiliations:** Université de Lorraine, INSERM, Centre d'Investigations Cliniques Plurithématique 1433, Inserm U1116, CHRU de Nancy and F-CRIN INI-CRCT, Nancy 54500, France; Department of Cardiology, Tokyo Medical University Hospital, Tokyo, Japan; Université de Lorraine, INSERM, Centre d'Investigations Cliniques Plurithématique 1433, Inserm U1116, CHRU de Nancy and F-CRIN INI-CRCT, Nancy 54500, France; Université de Lorraine, INSERM, Centre d'Investigations Cliniques Plurithématique 1433, Inserm U1116, CHRU de Nancy and F-CRIN INI-CRCT, Nancy 54500, France; TriNetX Europe NV, St.Martens-Latem, Belgium; TriNetX Europe NV, St.Martens-Latem, Belgium; Université de Lorraine, INSERM, Centre d'Investigations Cliniques Plurithématique 1433, Inserm U1116, CHRU de Nancy and F-CRIN INI-CRCT, Nancy 54500, France; Université de Lorraine, INSERM, Centre d'Investigations Cliniques Plurithématique 1433, Inserm U1116, CHRU de Nancy and F-CRIN INI-CRCT, Nancy 54500, France

**Keywords:** Heart failure, Heart failure and reduced ejection fraction, Prognosis, Sodium glucose co-transporter 2 inhibitor

## Abstract

**Introduction:**

Sodium-glucose cotransporter-2 inhibitors (SGLT2is) improve outcomes in patients with heart failure (HF) and are recommended to be initiated in the 6 weeks following an HF hospitalization. We aimed to explore prescription rates and clinical benefits of SGLT2is among patients with newly diagnosed HF and reduced ejection fraction (HFrEF) in real-world practice.

**Methods:**

We conducted a retrospective analysis using the TriNetX Global Collaborative research network. Patients with HFrEF who experienced their first HF hospitalization between September 2021 and December 2023 were identified and were categorized into two groups based on the initiation of SGLT2is within 6 weeks following HF hospitalization. After using propensity score matching to baseline characteristics, Cox hazard ratios (HRs) were calculated to compare outcomes over a 1-year period.

**Results:**

Among the identified 70 042 patients with HFrEF, 21.3% were initiated on SGLT2is within 6 weeks following their first HF hospitalization. Sodium-glucose cotransporter-2 inhibitor users were younger, more likely to be male, and had a higher prevalence of diabetes, compared with SGLT2i non-users. After matching, 14 670 matched pairs were created (mean age 64 ± 17 years; 41.6% female; 20% Black). Sodium-glucose cotransporter-2 inhibitor users vs non-users had a lower risk of 1-year all-cause mortality [HR, 95% confidence interval (CI) = 0.75, 0.69–0.83], all-cause hospitalizations (HR, 95% CI = 0.86, 0.83–0.91), and emergency department visits (HR, 95% CI = 0.91, 0.86–0.96).

**Conclusion:**

In this large multinational real-world data of patients with HFrEF, the prescription rate for SGLT2is within 6 weeks after the first HF hospitalization remained low. However, SGLT2i initiation was associated with improved outcomes, underscoring the importance of guideline-recommended early use.

## Graphical Abstract

**Graphical Abstract:**
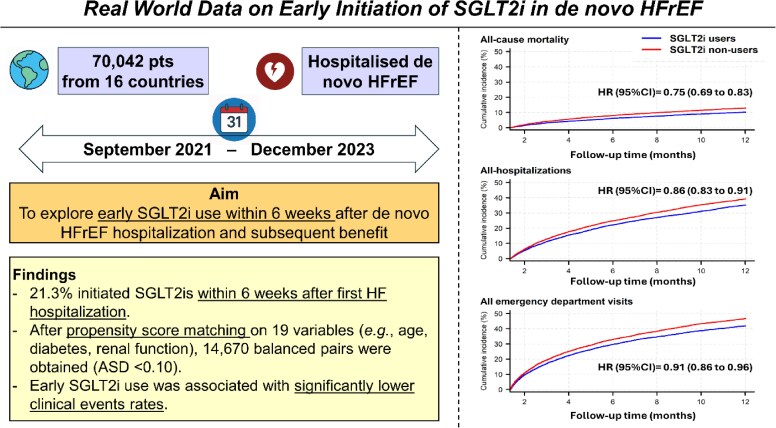

## Introduction

The latest guidelines strongly recommend use of sodium glucose co-transporter 2 inhibitors (SGLT2is) for the treatment of heart failure (HF), regardless of left ventricular ejection fraction (LVEF) and presence or absence of diabetes.^1,2^ In the EMPEROR-Reduced (Cardiovascular and Empagliflozin Outcome Trial in Patients with Chronic Heart Failure and a Reduced Ejection Fraction) and the DAPA-HF (Dapagliflozin and Prevention of Adverse Outcomes in Heart Failure) trials, SGLT2i empagliflozin and dapagliflozin reduced the composite risk of cardiovascular death or hospitalization for HF in patients with reduced ejection fraction (HFrEF).^3,4^

The updated European Society of Cardiology (ESC) guidelines recommended optimizing treatment before discharge and close follow-up visits in the first 6 weeks following a HF hospitalization.^5^ This Class I recommendation was supported by findings of the Safety, Tolerability and Efficacy of Rapid Optimization, Helped by NT-proBNP Testing, of Heart Failure Therapies (STRONG-HF) trial, which showed that intensified HF treatment up-titration vs usual care reduced the risk of all-cause mortality or HF hospitalization over 6 months.^6^ Additionally, a recent meta-analysis showed that SGLT2is reduced the early risk of all-cause mortality in patients hospitalized for HF.^7^ However, initiation of SGLT2is was frequently delayed, especially in patients with newly diagnosed HF, and overall prescription rates remained suboptimal in real-world practice.^8–10^

To address these potential uncertainties about the effectiveness of the early initiation of SGLT2is in real-world clinical practice, we investigated its effects on clinical outcomes in patients with *de novo* hospitalized for HF.

## Methods

### Study population

This study included individuals aged ≥18 years from 16 countries diagnosed with HFrEF, who experienced a first HF hospitalization requiring intravenous diuretic therapy within the preceding 6 weeks. Data were collected between September 2021 and December 2023 from the TriNetX Global Collaborative research network, which is a federated multicenter research network that provides real-time access to an anonymized data from participating healthcare organizations’ electronic health records (EHRs) as described in prior studies.^11,12^ Given regional variations in data types, new data are integrated into the TriNetX common data model for syntactic harmonization. Custom connectors, tools, and Application Programming Interfaces ingest data from various common models and healthcare exchange standards. Semantic harmonization is accomplished by mapping data to TriNetX terminology standards, which are selected to reflect typical domain-specific data capture [e.g. International Classification of Diseases (ICD) for diagnoses] and reduce mapping burden. Medication terminologies are updated quarterly, while other standards are refreshed annually. Unmapped concepts are tracked, regularly reviewed, and compared against the latest standards.^13^

A diagnosis with HFrEF was based on ICD-9th Revision and ICD-10th Revision, Clinical Modification code 150.2 (i.e. 428.20, 428.21, 428.22, 428.23). Patients were further divided into two groups based on whether they were treated or not with SGLT2is (dapagliflozin or empagliflozin) within 6 weeks following an HF hospitalization.

### Statistical analysis

Categorical variables are presented as frequencies (percentages) and continuous variables as means ± standard deviations (SDs). Comparisons of baseline characteristics between two treatment groups were analysed using the independent-samples Student’s *t*-tests and *χ*^2^ test. All covariates are presented in *Table 1* and were matched extensively by 1:1 propensity score matching method using absolute standardized difference (ASD). The ASD quantifies the difference between the means of two groups, SGLT2i users and non-users, regarding SD units to assess the balance of baseline variables in the sample weighted by the inverse probability of treatment. The ASD of any baseline characteristics lower than 0.10 was considered well-matched.

**Table 1:** Baseline characteristics before and after propensity score matching

	Before matching				After matching			
	SGLT2i users (*N* = 14,923)	SGLT2i non-users (*N* = 55 119)	*P*-value	Absolute standardized difference	SGLT2i users (*N* = 14 670)	SGLT2i non-users (*N* = 14 670)	*P*-value	Absolute standardized difference
Age, years	63.8 ± 14.5	68.2 ± 16.1	<.001	0.287	64.0 ± 14.4	64.1 ± 16.7	.658	0.005
Male, ***N* (%)**	9556 (64.0%)	32 053 (58.2%)	<.001	0.121	9364 (63.8%)	9438 (64.3%)	.368	0.011
Race, ***N* (%)**								
White	9060 (60.7%)	36 916 (67%)	<.001	0.131	8957 (61.1%)	9002 (61.4%)	.590	0.006
Black	3066 (20.5%)	8974 (16.3%)	<.001	0.110	2968 (20.2%)	2927 (20.0%)	.550	0.007
Medical history, ***N* (%)**								
Hypertension	7764 (52.0%)	29 909 (54.3%)	<.001	0.045	7636 (52.1%)	7520 (51.3%)	.175	0.016
Diabetes mellitus	6893 (46.2%)	21 986 (39.9%)	<.001	0.128	6728 (45.9%)	6766 (46.1%)	.656	0.005
Ischaemic heart disease	8688 (58.2%)	29 868 (54.2%)	<.001	0.081	8527 (58.1%)	8480 (57.8%)	.578	0.006
Myocardial infarction	4855 (32.5%)	17 351 (31.5%)	.014	0.023	4776 (32.6%)	4770 (32.5%)	.940	0.001
Atrial fibrillation	5808 (38.9%)	24 515 (44.5%)	<.001	0.113	5735 (39.1%)	5738 (39.1%)	.971	<0.001
Overweight/obesity	4483 (30.0%)	14 656 (26.6%)	<.001	0.077	4380 (29.9%)	4460 (30.4%)	.309	0.012
COPD	4038 (27.1%)	16 720 (30.3%)	<.001	0.072	3983 (27.2%)	3999 (27.3%)	.834	0.002
Chronic kidney disease	4092 (27.4%)	20 128 (36.5%)	<.001	0.196	4057 (27.7%)	3920 (26.7%)	.072	0.021
Cerebrovascular disease	1947 (13.0%)	10 143 (18.4%)	<.001	0.148	1937 (13.2%)	1871 (12.8%)	.252	0.013
Medication, ***N* (%)**								
Beta-blocker	10 025 (67.2%)	37 952 (68.9%)	<.001	0.036	9830 (67.0%)	9792 (66.7%)	.637	0.006
ACEi	3382 (22.7%)	12 368 (22.4%)	.56	0.005	3333 (22.7%)	3265 (22.3%)	.342	0.011
ARB	5866 (39.3%)	14 326 (26%)	<.001	0.287	5620 (38.3%)	5560 (37.9%)	.471	0.008
ARNI	2804 (18.8%)	3737 (6.8%)	<.001	0.366	2564 (17.5%)	2488 (17.0%)	.24	0.014
MRA	3601 (24.1%)	7190 (13.0%)	<.001	0.288	3385 (23.1%)	3365 (22.9%)	.781	0.003
Loop diuretic	14 913 (99.9%)	55 119 (100%)	<.001	0.037	14 669 (100%)	14 670 (100%)	.317	0.012
Laboratory								
Creatinine, mg/dl	1.5 ± 5.5	1.7 ± 4.2	<.001	0.041	1.5 ± 5.6	1.3 ± 2.6	<.001	0.046
≤1.20 mg/dl	10 300 (69.0%)	35 651 (64.7%)	<.001	0.092	10 130 (69.1%)	10 199 (69.5%)	.383	0.010
1.20–1.50 mg/dl	5161 (34.6%)	19 275 (35%)	.381	0.008	5074 (34.6%)	4960 (33.8%)	.161	0.016
1.50–2.00 mg/dl	3328 (22.3%)	14 735 (26.7%)	<.001	0.103	3299 (22.5%)	3243 (22.1%)	.432	0.009
2.00–3.00 mg/dl	1598 (10.7%)	9929 (18.0%)	<.001	0.209	1594 (10.9%)	1570 (10.7%)	.651	0.005
>3.00 mg/dl	577 (3.9%)	6842 (12.4%)	<.001	0.316	577 (3.9%)	554 (3.8%)	.486	0.008

Values are mean ± SD, *n* (%) or median (25th–75th percentile).

SGLT2i, sodium glucose co-transporter 2 inhibitor; COPD, chronic obstructive pulmonary disease; ACEi, angiotensin converting enzyme inhibitor; ARB, angiotensin receptor blocker; ARNI, angiotensin receptor neprilysin inhibitor; MRA, mineralocorticoid receptor antagonist.

Primary and secondary outcomes were analysed over a 1-year follow-up period. The primary outcome was all-cause mortality. Other secondary outcomes included all-cause hospitalizations and emergency department visits. Time-to-event comparisons were analysed using log rank test and Cox proportional hazards models. Survival probabilities were estimated using the Kaplan–Meier method and plotted as survival curves between treatment allocations.

Statistical analyses were performed based on the TriNetX online platform using R for statistical computing. A two-sided *P*-value <.05 was considered statistically significant.

## Results

### Baseline characteristics

Baseline patient characteristics are presented in *Table 1*. Among 70 042 patients newly diagnosed with HFrEF, 14 923 patients were initiated with SGLT2is (empagliflozin 53%; dapagliflozin 47%) within 6 weeks from their first HF hospitalization, while 55 119 patients did not receive this treatment.

Sodium-glucose cotransporter-2 inhibitor (SGLT2i) users within 6 weeks were younger, more likely to be male, and Black or African American, compared with SGLT2i non-users (all-*P* < .001). Furthermore, SGLT2i users had a higher prevalence of diabetes mellitus and ischaemic heart disease, but a lower prevalence of chronic kidney disease (CKD), and were more often treated with sacubitril-valsartan, compared with SGLT2i non-users (all-*P* < .001) (*Table 1*).

Following the matching process, 14 670 patients in each SGLT2i user and non-user group were retained and were included in the present analysis. The baseline characteristics of the two groups were well-balanced (standardised mean difference <0.1 for all covariates). Mean age was 64 ± 17 years, 64.3% of matched participants were females, 46.1% had diabetes, 57.8% had ischaemic heart disease, and 26.7% had CKD. For HF treatments, 77.2% of patients were on angiotensin-converting enzyme inhibitor (ACEi), angiotensin receptor blocker (ARB), or angiotensin receptor-neprilysin inhibitor (ARNI), 66.7% were on β-blockers and 22.9% were on mineralocorticoid receptor antagonists (MRAs) (*Table 1*).

### Clinical outcomes

**Figure 1:**
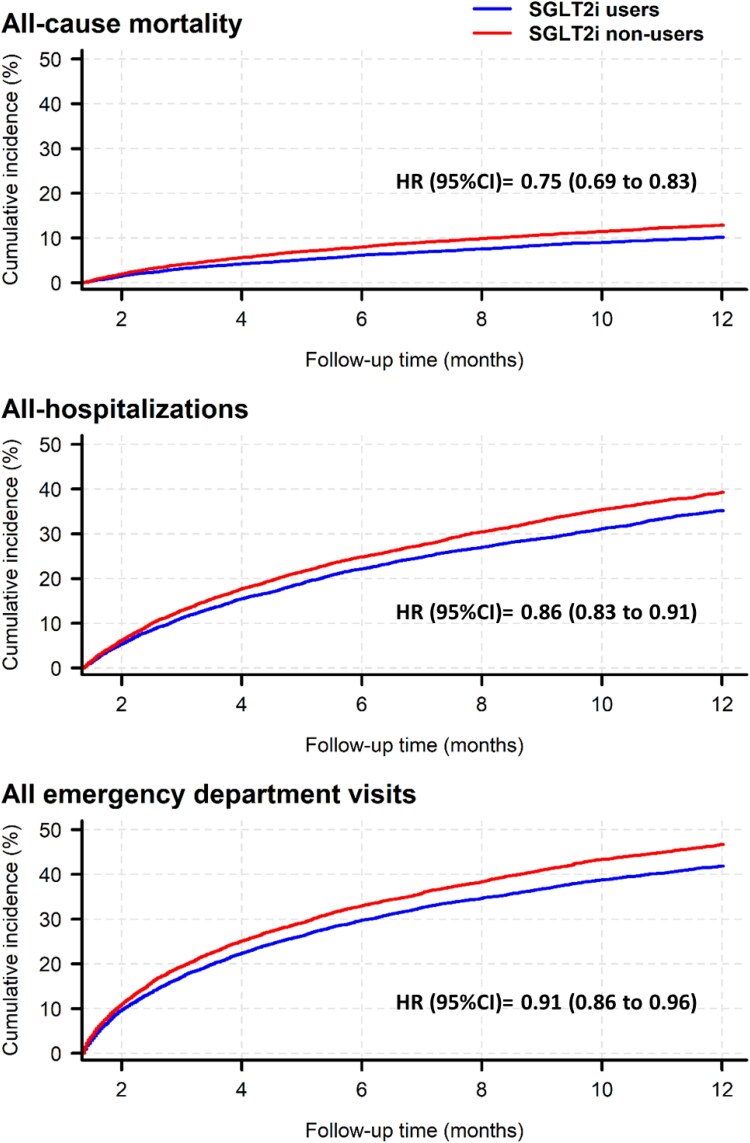
Survival curves for the primary endpoint of all-cause mortality, and secondary endpoints of all-cause hospitalizations and emergency department visits. SGLT2i, sodium glocose co-transporter 2 inhibitor; HR, hazard ratio; CI, confidence interval

All-cause mortality was observed in 19.4% of SGLT2i users compared with 24.7% of SGLT2i non-users [hazard ratio (HR), 95% confidence interval (CI) = 0.75, 0.68–0.83; *P* < .001). Early initiation of SGLT2i after a first HF hospitalization reduced the risk of all-cause hospitalization (HR, 95% CI = 0.86, 0.83–0.91) and all-cause emergency department visits (HR, 95% CI = 0.91, 0.86–0.96; *Figure 1*).

In a landmark analysis at 6 months, risk of all-cause mortality did not differ significantly between patients who initiated SGLT2i within 6 weeks and those who did between 6 weeks and 6 months after hospitalization (HR, 95% CI = 0.94, 0.82–1.07).

**Figure 2:**
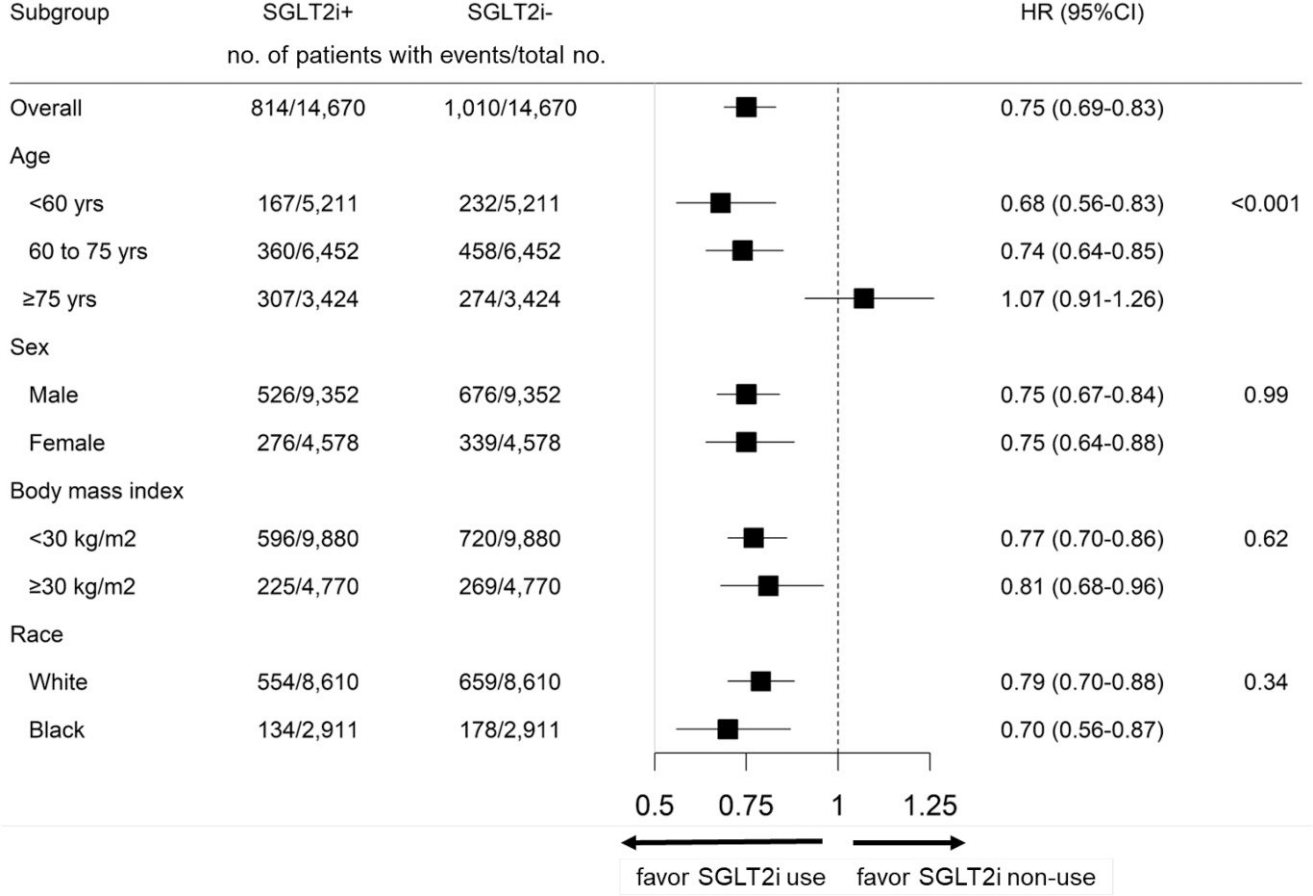
Subgroup analysis for all-cause mortality. SGLT2i, sodium glocose co-transporter 2 inhibitor; HR, hazard ratio; CI, confidence interval

For subgroups analysis, the efficacy of SGLT2is on mortality was consistent regardless of sex, obesity, and racial disparity (*P*-for-interaction >.10). However, among patients under 75 years of age, the benefit from SGLT2is was more pronounced, while in those aged 75 or older, the impact on all-cause mortality was neutral (HR, 95% CI = 1.07, 0.91–1.26; interaction *P*-value <.001; *Figure 2*).

## Discussion

In this large multinational real-world data network, we found that the initiation of SGLT2is within 6 weeks following HF hospitalization was infrequent among patients with newly diagnosed HFrEF. However, early initiation of SGLT2is reduced the risk of all-cause mortality by 25%, hospitalization for any causes by 10%, and emergency department visit by 14%. The STRONG-HF trial, which supported the rapid initiation of HF treatments following a hospitalization in the latest guidelines,^5^ primarily focused on conventional HF treatments (i.e. ACEi/ARB/ARNI, β-blocker, and MRA), while a limited number of patients were treated with SGLT2is per the study protocol (<10%). Nonetheless, more rapid efficacy of SGLT2is was evidenced in clinical trials for chronic HFrEF.^14,15^ Collectively, our findings suggest that the early initiation (<6 weeks) of SGLT2is, as recently supported by the focused update of the ESC guidelines,^5^ could significantly benefit clinical outcomes in patients with newly diagnosed HFrEF.

Patients included in the present analysis had relatively low prescription rates of HF treatments as well as a low risk of mortality. Our study specifically focused on patients with newly diagnosed HF, potentially explaining these observed low rates of life-saving drug use and mortality risk.^16–18^ Furthermore, different practice patterns, in-hospital and post-discharge managements and health insurances across countries may influence the prognosis of patients hospitalized for HF.^19,20^ However, this unique analysis through the TriNetX network unlocks access to global data, covering over 16 countries, thereby capturing the pattern of therapeutic management on a global scale.

Interestingly, our results showed a greater reduction in all-cause mortality by 25% with SGLT2i treatment in patients with HFrEF, which was higher than those from prior published data across clinical trials for SGLT2is in HF, CKD, and type 2 diabetes (<10% reduction of all-cause mortality).^3,4,21–26^ Our study included patients hospitalized for HF, whose prognosis were generally more likely to be impacted by the congested status. The mechanisms, therefore, of the prominent effects may be underpinned by natriuretic or diuretic effects of SGLT2is in the relatively early phase after its initiation.^27–29^ In the Study to Test the Effect of Empagliflozin in Patients Who Are in Hospital for Acute Heart Failure (EMPULSE) trial including 530 patients with hospitalized HF, empagliflozin vs placebo reduced all-cause mortality, HF events and symptoms at 90 days.^30^ In the Effect of Sotagliflozin on Cardiovascular Events in Patients with Type 2 Diabetes Post Worsening Heart Failure (SOLOIST-WHF) trial including 1222 patients with recently hospitalized HF and Type 2 diabetes, sotagliflozin vs placebo reduced cardiovascular deaths, hospitalizations and urgent visits for HF over the 9.0-month median follow-up.^31^ Furthermore, a meta-analysis of these two trials together with the Dapagliflozin Effect on Cardiovascular Events in Acute Heart Failure-Thrombolysis in Myocardial Infarction 68 (DAPA ACT HF-TIMI 68) trial showed that SGLT2i reduced mortality risk by 43%, supporting the efficacy of SGLT2i observed in our real-world data.^7^ Additionally, the observed reductions in hospitalization or emergency department visit suggests a potential benefit of SGLT2is in preventing subsequent life-threatening events in individuals with HFrEF, which align with the EMPEROR-reduced and DAPA-HF trials.^15,32^ Interestingly, our landmark analysis showed that long-term outcomes appeared similar once SGLT2i therapy was eventually initiated. However, even if long-term outcomes converge, delays in SGLT2i initiation can still expose patients to preventable adverse events during the vulnerable post-hospitalization phase (i.e. within the first 6 months).

Our results from large-sized data may also address several key uncertainties regarding the potential efficacy of SGLT2i in under-represented subgroups of patients with HFrEF such as women and Black patients.^33^ Although these clinical presentations may influence an HF risk and the effects of certain HF treatments,^34–36^ we observed no significant heterogeneity in the clinical benefits of SGLT2is across different sex, body mass index, and racial categories. These findings are supported by prior published data from SGLT2i trial for chronic HFrEF^37–40^ and provide the robust evidence regarding the initiation of SGLT2is within 6 weeks following a first HF hospitalization. Additionally, our analysis showed that older patients experienced diminished mortality benefits from SGLT2is, likely reflecting both biological factors (e.g. frailty, multimorbidity, and impaired drug metabolism) and practical barriers (e.g. suboptimal dosing, polypharmacy, reduced adherence, and limited monitoring).^41^

Randomized clinical trials showed in-hospital initiation of SGLT2i to be safe and provide significant early post-discharge benefits on patient symptoms and quality of life.^30,31^ Taken together, the present study is the first global study addressing the real-world effectiveness of SGLT2i in a very large number of patients with newly diagnosed HFrEF. These findings highlight the merits of early SGLT2i initiation and further support current recommendations advocating early initiation of guideline-directed therapies in patients hospitalized or recently discharged following acute HF.^42,43^

### Limitations

The results presented should be interpreted within the context of several potential limitations. This retrospective study was observational in nature, and despite robust propensity score matching, the possibility of residual or unmeasured confounding cannot be excluded. Differences in healthcare systems, patient socioeconomic status, and clinical practice patterns across 16 participating countries, as well as unmeasured clinically relevant variables (e.g. natriuretic peptide levels, LVEF and doses of ACEi/ARB, ARNI, or β-blocker) may have influenced our results. Although we focused on patients with newly diagnosed HF, this study included those with relatively low prescription rates of HF treatments as well as a low mortality risk.^16–18^ An EHR-based data network is susceptible to errors in coding, particularly for HFrEF diagnosis. Additionally, outcomes that occurred outside of the TriNetX network were not well captured. We lack specific information on the causes of death, hospitalization, and emergency department visits; however, patients with hospitalized HFrEF, generally, tend to experience HF-related events rather than non-cardiac causes during the 1-year post-discharge period.^44,45^ Nonetheless, the present analysis included a substantially large number of patients, potentially sufficient to mitigate the effects of ‘random noise’; for example, stemming from non-cardiovascular deaths.^46^

## Conclusions

In this large multinational real-world data of patients with HFrEF, the prescription rate for SGLT2is within 6 weeks after the first HF hospitalization remained limited. However, SGLT2i initiation during this period was associated with improved clinical outcomes. These findings highlight the importance of early initiation, as endorsed by the latest guidelines.

## Declarations

### Data Availability

All data used in the generation of this paper was collected from the global TriNetX network. The data that support the findings of this study are available from TriNetX, LLC, https://trinetx.com/, but third-party restrictions apply to the availability of these data. The data were used under licence for this study with restrictions that do not allow the data to be redistributed or made publicly available. However, for accredited researchers, the TriNetX data are available for licencing at TriNetX, LLC. Data access may require a data sharing agreement and may incur data access fees.

### Pre-registered Clinical Trial Number

None supplied.
